# The antibacterial mechanism of compound preservatives combined with low voltage electric fields on the preservation of steamed mussels (*Mytilus edulis*) stored at ice-temperature

**DOI:** 10.3389/fnut.2023.1126456

**Published:** 2023-03-17

**Authors:** Kunmei Wang, Han Wang, Yue Wu, Chong Yi, Yanxia Lv, Hongyu Luo, Tao Yang

**Affiliations:** ^1^Zhejiang Provincial Key Laboratory of Health Risk Factors for Seafood, Collaborative Innovation Center of Seafood Deep Processing, College of Food and Pharmacy, Zhejiang Ocean University, Zhoushan, China; ^2^Yantai Marine Economic Research Institute, Yantai, China

**Keywords:** compound preservative, low voltage variable frequency electric field, *Bacillus subtilis*, *Pseudomonas*, steamed mussels

## Abstract

Mussels are a kind of economically valuable ocean bivalve shellfish. It has a short harvest period and is susceptible to contamination during storage and processing. Having proper preservation methods is critical to prevent quality deterioration. However, the effect of low voltage variable frequency electric field and compound preservative on the freshness of steamed mussels in ice-temperature storage are still unknown. We utilized the method of coefficient variation weighting to calculate the overall scores of steamed mussels stored under different preservation conditions. The protein physicochemical properties of samples, the growth curves of two dominant spoilage bacteria; *Bacillus subtilis* and *Pseudomonas* in the mussels as well as the Structural changes of the cell membranes were mensurated. The results show that compared with the preservative group and the low voltage variable frequency electric field group, the compound preservatives combined with the electric field group had the highest overall score and thus the best preservation effect. Compared with the blank group, the total sulfhydryl content and myogenic fibrin content of the combined group decreased at the slowest rate, 19.46%, and 44.92%, respectively. The hydrophobicity of the protein surface increased by only 5.67%, with the best water retention, indicating that the samples of the combined group had the least protein deterioration in the combined group. The inhibition mechanism of the combined group inhibited the growth of two dominant spoilage bacteria: *Bacillus subtilis* and *Pseudomonas*, in the mussels, destroying the integrity of the cell membrane structure and changing the cell morphology. Overall, we found that the combination of the composite preservatives and the low voltage variable frequency electric field can maintain the best quality of steamed mussels during ice-temperature storage and slow down the rate of protein deterioration during storage. This study proposed a new method of mussel preservation, which provides a new idea for the application of low voltage variable frequency electric field and compound preservative in the preservation of aquatic products.

## 1. Introduction

Mussels are a kind of bivalve edible shellfish with fast growing, seasonal and productive (1). Mussel meat is rich in proteins, sugars, minerals, vitamins, and amino acids and thus has high nutritional values (2). Due to the high moisture and protein content of mussels, mussel harvesting season is concentrated and the harvesting period is short, the mussels easily rot and deteriorate mussels if they are stored or processed improperly (3, 4). At present, in addition to fresh sales, mussels are mainly processed into semi-finished frozen products for cooking or dry products for longer storage. If the quality of storing steamed mussels can be mastered, and challenges with preserving them be well solved, it will not only allow for mussels to be processed outside of the harvesting season, but it will also guarantee new developments for processing mussel products from a raw materials perspective. Therefore, the preservation technology of mussels is very important to break through the bottleneck of the mussel industry.

In recent years, through the in-depth study of electromagnetism, domestic and foreign experts have gradually carried out the application of electric fields in food industrial production. Different from direct current electric fields, low voltage variable frequency electric fields (LVVFEF) are space electric fields with 50 Hz as the frequency needed to change the direction of electric fields and the electric field strength is no more than 3,000 V (5). By periodically changing the direction of the electric field, a LVVFEF equalizes the electric field force within the food, resulting in strong vibrations (6). This improves the time required for food to pass the maximum ice crystal formation and the freezing rate to a certain extent. LVVFEF have many applications in food preservation, as it assists with accelerating the freezing and thawing process, preserving frozen foods, food sterilization, and so on. At the same time, LVVFEF also influences the enzyme activity and biological effects in an organism. Mousakhani et al. (7) used an electric field to thaw tuna. The use of the electric field not only increased the thawing rate but also reduced the level of volatile salt nitrogen in the tuna. Ultimately, there was no significant difference between the thawed tuna and fresh fish in elasticity and chewability. Ko et al. (8) added tilapia fillets to high voltage electric field (HVEF) at 600 kv/m and 900 kv/m, which not only inhibited ATP degradation, protein loss, and microbial growth but it also effectively prolonged the shelf life of the fish. Bai et al. (9) showed that HVEF can inhibit the growth of bacteria in *Scomberomorus niphonius* during storage, reduce TVB-N, and prolong the storage period. According to Zituni et al. (10), the growth of the bacteria *Staphylococcus aureus* was found to be significantly inhibited when it was exposed to an electric field incubation with an intensity of 4–10 V/m.

When preservatives are used for food storage and preservation, they may be limited by their own antibacterial spectrum, physical and chemical properties, and other factors. Lan et al. (11) showed that pectin combined with plant essential oils can effectively prolong the storage period of large yellow croaker in ice temperature environment. According to the principle of fence technology, using different sources of preservatives at the same time can play a synergistic effect, enhance the preservation effects, and prolong shelf life. It has been reported that *Bacillus subtilis* and *Pseudomonas* are the main dominant bacteria leading to mussel spoilage to the principle of fence technology (12). Additionally, the principle of fence technology utilized in previous research has found that biopreservatives compounded by *Lysozyme*, *Nisin,* and E*ugenol* had good antibacterial effects on these two bacteria(*Bacillus subtilis* and *Pseudomonas*). Other studies have also explored the effects of LVVFEF on the quality of steamed mussels during ice-temperature storage. However, the effects of LVVFEF combined with compound preservatives on the preservation of steamed mussels at freezing temperature and the degree of protein deterioration delay as well as the antibacterial mechanisms are still unclear. This paper aims to fill this gap by investigating how LVVFEF joined with compound preservatives influence points of frozen storage for steamed mussels at the end of pH and TVB-N. We also explore the total number of colonies and sensory evaluation of stored mussels, as well as the influence of simultaneous determination of mussel protein deterioration using a storage characteristic index. The effects on the structural properties of the cell membranes of two dominant bacteria were investigated to examine the mechanisms of preservation and bacterial inhibition in this combined preservation mode by comparing results with control groups and single-factor effect groups.

## 2. Materials and methods

### 2.1. Materials and experimentation

Mussels were purchased from Zhoushan International Fisheries City (Zhejiang Province, China). The selected mussels had a full and well-proportioned body shape and were odor-free. All samples were packed in a box filled with water containing dissolved oxygen and shipped to the laboratory of Zhejiang Ocean University. Once arrival, all the fresh mussels were washed, spit sand for 24 h, and boil in put water at 100°C for 15 min. Collect the cooked mussel meat on a sterile workbench, place in a clear polyethylene bagged and sealed. The samples were randomly divided into four groups; the control group (CG), the compound preservative group (CPG), the low voltage variable frequency electric field group (LEG), and the compound preservative combined with low voltage variable frequency electric field group (CP+LEG). The sample group was placed 15 cm away from an electric field plate. By measuring the inflection point of the mussel freezing curve and the temperature fluctuation of the freezer, we found that the optimal ice temperature storage temperature of the steamed mussels was −1 ± 0.5°C. The remainder of this paper is divided into two parts; the first part is a comprehensive scoring of the quality of each group of samples by measuring the physicochemical indexes of the samples and using the weight coefficient method. The second part investigates the mechanistic investigation of the preservation effects of steamed mussels by determining the physicochemical indexes of protein deterioration and investigating the mechanisms of bacterial inhibition.

### 2.2. Treatment conditions of the experimental group

CG: This group was cooked only and no other treatment was given.

CPG: Based on the results of the optimization of the formulation of the composite preservatives for steamed mussels by our team Lv et al. (13), the sample in this group was immersed in the composite preservation solution (4% *Lysozyme* + 2% *Nisin* + 0.75% *Eugenol*) at a solid–liquid ratio of 1:2.5 for 60 min, and then stored in a − 1 ± 0.5°C refrigerator.

LEG: This sample was placed in a refrigerator equipped with a discharge board. The sample was 15 cm away from the discharge board, the electric field strength was 3,000 V, the electric field frequency was 50 Hz, and the refrigerator temperature was adjusted to −1 ± 0.5°C.

CP+LEG: This sample was first immersed in a composite fresh-keeping solution, and then treated by an electric field. The treatment conditions were the same as those of the compound preservative group and the electric field group.

### 2.3. Physicochemical parameters

#### 2.3.1. pH

The assay was performed by referring to the method of Violetta et al. (14) with slight modifications. The pH of the homogenized samples (10 g in 100 mL of peptone diluent) was measured with a pH meter(PHS-3C Thunder Magnetic bench pH meter: Shanghai Yidean Scientific Instruments Co., LtD., China).

#### 2.3.2. The total number of colonies

The total number of colonies was determined following the GB 4789.2–2016 standard. The samples were diluted with physiological saline 1:9 (m/V), homogenized, and then serially diluted 10-fold. Two to three suitable dilutions were selected and added to the plate count agar (PCA) agar medium. The PCA medium was purchased from Hangzhou Microbial Reagent Co. Ltd. (Hangzhou, China), and after the agar solidified, the Petri dishes were inverted and incubated under the corresponding conditions. The total number of colonies was counted after incubation at 30 ± 0.5°C for 48 h. The results were expressed as g (CFU/g).

#### 2.3.3. TVB-n

TVB-N content was determined according to the automatic Kjeldahl method as per the GB 5009.228–2016 standard. TVB-N was measured using an automatic Kjeldahl apparatus (JK9870; Focus Technology Co., Ltd., China). Ten grams of samples were homogenized with 75 mL distilled water and soaked for 30 min. TVB-N content was expressed as mg/100 g.

#### 2.3.4. Sensory evaluation

The sensory evaluation was based on SC/T 3209–2012 “Mussels” standard with slight modifications of Table 2-4 standards. A sensory assessment team composed of 10 trained personnel evaluated and scored the color, tissue morphology, and odor of the samples according to the criteria.

#### 2.3.5. Comprehensive evaluation method for mussel quality

The comprehensive evaluation method for mussel quality was evaluated according to the method described by Niu (15) with some modifications. The coefficient of variation weighting method was used to comprehensively evaluate the quality differences of mussels under different electric field intensities. Firstly, the coefficient of variation of each index is obtained by calculating the arithmetic mean and standard deviation of each index, and then the weight value of each index is obtained by using the coefficient of variation, and all the data are standardized accordingly, and the comprehensive score of mussels under different electric field treatment conditions is obtained by the weighted average method.

### 2.4. Total sulfhydryl content

A total sulfhydryl measurement kit (Nanjing Jiancheng Bioengineering Institute, China) was used to measure the total sulfhydryl content. The assay was performed by referring to the method of Sun et al. (16) with slight modifications. 2.0 g of the sample was placed into a 50 mL centrifuge tube and 10 mL of 50 mmol/L phosphate buffer (pH 8.0) was added to it. It was homogenized in the centrifuge with 1 mL of the supernatant. A 10-fold dilution with distilled water was also added, then 20 μL of 2 mmol/L DTNB reagent was added and mixed *via* a vortex. We measured the absorbance at 412 nm after 1 h of shading at 25°C. A BCA kit was used to determine the protein concentration. The total sulfhydryl content was calculated according to Equation 1. The results of the total SH content were expressed as μmol/g prot.


(1) $$\mathrm{Total sulfhydryl content}\;\left(\mu \mathrm{mol}/g\;\mathrm{prot}\right)=\frac{A\times B}{C\times D}$$

### 2.5. Protein surface hydrophobicity

The protein surface hydrophobicity was evaluated according to the method described by Liu (17) with some modifications. Determination was performed by the bromophenol blue (BPB) method. One mL of protein solution was distributed into the centrifuge at 10,000 r/min for 5 min. The supernatant was poured in and 1 mL of PBS buffer was added at 20 mmol/l pH 6.0. 80 μL of bromophenol blue (BPB, 1 mg/mL) was added and stirred for 10 min at room temperature. Then, the solution was centrifuged at 10,000 r/min for 15 min in centrifuge. The supernatant (after appropriate dilution) was taken and the absorption value was measured at A1 at an absorbance of 595 nm. The bromophenol blue blank sample was 200 μL of bromophenol blue with 1 mL of pH 6.0, 20 mmol/l PBS buffer, and the absorbance A_0_ was determined under the same conditions. The surface hydrophobicity was calculated according to Equation 2.


(2) $$\mathrm{Bromophenol blue binding amount}\;\left(\mathrm{\mu g}\right)=\frac{200\times \left(A0-A1\right)}{Α0}$$

### 2.6. Preparation of bacterial suspension

The experimental indicator strains *Bacillus subtilis* and *Pseudomonas* were inoculated in the nutrient broth medium, incubated at 180 r at 30°C for 12 h, and then inoculated in the new nutrient broth with 1% inoculum until the OD value was between 0.5 and 0.6, that is, the final concentration of bacterial suspension was about 107 CFU/mL.

### 2.7. Determination of thallus growth curve

Referring to the method of Shu et al. (18) with the appropriate modifications, we explored the effects of compound preservatives combined with a LVVFEF on the growth of *Bacillus subtilis* and *Pseudomonas*. In this experiment, the growth curves of Bacillus species under different conditions were investigated (Table 1). The samples of each group were cultured for 24 h. The absorbance of the strains was measured at 600 nm wavelength by a microplate reader every 1 h, and the growth curve was drawn.

**Table 1:** Grouping of *Bacillus subtilis* and *Pseudomonas* growth curves.

CG	*Bacillus subtilis*/ *Pseudomonas* NG	*Bacillus subtilis*/ *Pseudomonas* MIC	*Bacillus subtilis*/ *Pseudomonas* LEG
200 μL Distilled water	190 μL bacteria suspension and	190 μL bacteria suspension	190 μL bacteria suspension and
	10 μL LB-Broth-Medium	10 μL of compound preservative with MIC concentration	10 μL LB-Broth-Medium
Culture temperature 37°C			Electric field parameters: 3000 V, 50 Hz culture temperature 37°C

MIC, minimum inhibitory concentration; The MICs of Bacillus subtilis and Pseudomonas were 16 μg/mL and 8 μg/mL, respectively.

### 2.8. Determination of bacterial cell membrane structure

To explore the inhibition mechanisms of the LVVFEF and compound preservatives on bacterial somatic cells, this paper refers to Yang (19), Shi (20), and Li (21). The permeability, membrane potential, and membrane integrity of the cell membranes were investigated (Table 2).

**Table 2:** Somatic cell membrane-related experiments.

Project	CG	MIC CPG	LEG	CP+LEG	Determination method
Permeability of cell membrane	5 mL bacteria suspension	4 mL bacterial suspension and 1 mL compound preservative	5 mL of bacterial suspension in an incubator equipped with a discharge plate	4 mL of bacterial suspension and 1 mL of compound preservative in an incubator equipped with a discharge plate	The culture was incubated at 37°C for 6 h, and the conductivity of the culture medium was measured every 1 h
Cell membrane potential	200 μL bacteria suspension	100 μL of bacterial suspension and 100 μL of preservative	200 μL of bacterial suspension was placed in an electric field incubator	100 μL of bacterial suspension and 100 μL of the preservative solution was placed in an electric field incubator	Each group was incubated for 2 h under specific conditions, then 1 μmol/LDiBAC4 (3) was added and mixed, and then incubated in an incubator without an electric plate at 37°C for 30–60 min in the dark, and the fluorescence intensity was detected
Nucleic acid leakage from the cell	200 μL bacteria suspension	190 μL bacterial suspension and 10 μL preservative solution	200 μL of bacterial suspension was placed in an electric field incubator	190 μL of bacterial suspension and 10 μL of preservative solution were placed in an electric field incubator	After culture at 37°C for 4 h, the absorbance was measured at 260 nm

### 2.9. Scanning with an electron microscope

Referring to the method of Lin et al. (22) with the appropriate modifications, cultured *Bacillus subtilis,* and *Pseudomonas* were attached to a coverslip to make a climbing sheet. The colonies were covered with the coverslip and 1 mL of the preservative solution was added to the CPG. This placed it in an environment with and without the electric field device. Untreated bacteria were used with the CG and incubated in an incubator for 12 h. Then, it was fixed in the fixing solution for 2 h at 4°C. The fixed thalli were washed three times with the PBS buffer. The treated samples were placed in the fixation solution for scanning under an electron microscope.

### 2.10. Data processing

Each group was measured three times in parallel, and the data were expressed as the mean ± standard deviation. Origin2022 software was used for plotting, and one-way analysis of variance (LSD) was used to evaluate the significance of differences among groups. *p* < 0.05 was considered a significant difference, and *p* > 0.05 was not a significant difference. SPSS 21.0 statistical analysis software was used to process the experimental data.

## 3. Results and discussion

### 3.1. Influence of preservation methods on the comprehensive score quality of steamed mussels

The freshness and quality of samples were characterized by many indexes. It is difficult to make an objective and scientific evaluation of the fresh-keeping effect by using a single index. Therefore, this paper adopted the coefficient of variation weighting method to analyze the contribution of different indexes to product freshness and quality, to determine the weight coefficient and corresponding standardized value of each index, and finally calculated the comprehensive score. The results are shown in Table 3.

**Table 3:** Comprehensive scores of the ice-temperature storage quality of steamed mussels.

Index	Weight factor	Date	Sample			
			CG	CPG	LEG	CP+LEG
pH	0.026	Average value	5.97 ± 0.01	5.85 ± 0.015	5.81 ± 0.014	5.79 ± 0.05
		Normalized values	−1.375	0.125	0.625	0.875
		Comprehensive	−0.035	0.003	0.016	0.022
TVB-N	0.682	Average value	16.7 ± 1.10	10.15 ± 0.35	8.73 ± 0.26	7.91 ± 0.21
		Normalized values	−1.461	0.18	0.536	0.742
		Comprehensive	−0.996	0.123	0.366	0.506
Total number of colonies	0.119	Average value	6.54 ± 0.06	5.84 ± 0.06	5.78 ± 0.04	5.72 ± 0.03
		Normalized values	−1.500	0.342	0.500	0.658
		Comprehensive	−0.179	0.041	0.060	0.078
Sensory score	0.174	Average value	19.61 ± 0.15	23.10 ± 0.17	23.83 ± 0.15	24.30 ± 0.25
		Normalized values	−1.462	0.184	0.528	0.750
		Comprehensive	−0.254	0.032	0.092	0.130
Comprehensive			−1.464	0.199	0.533	0.737
Sort			4	3	2	1

According to Table 3, the weighted coefficient of TVB-N is the largest, and the weight coefficient of pH is the smallest. This indicates that the preservation method has the greatest influence on TVB-N and the least influence on the pH of mussels stored at ice temperature. According to the total score of the comprehensive evaluation of each group, the compound preservative combined with a LVVFEF had the best effect on the preservation quality of steamed mussels at cold temperatures.

### 3.2. Total sulfhydryl content of mussels

**Figure 1:**
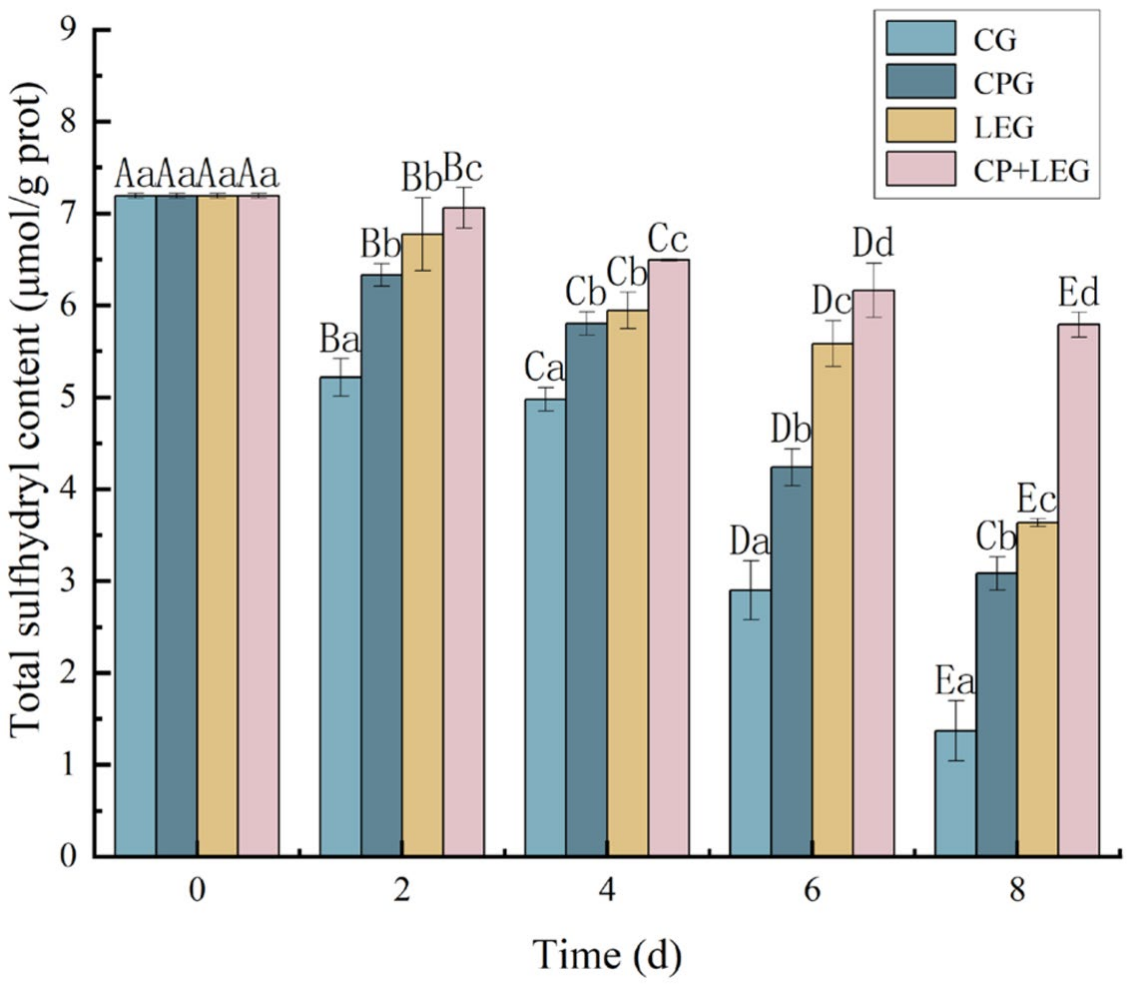
The effects of preservation methods on the total sulfhydryl content of mussels. CG, the control group; CPG, the compound preservative group; LEG, the low voltage variable frequency electric field group; CP+LEG, the compound preservative combined with low voltage variable frequency electric field group. At the same storage time, different lowercase letters indicate significant differences between groups; Within the same processing group, different uppercase letters indicate significant differences within the group (*p* < 0.05). Data were obtained as the mean ± standard deviation (*n* = 3).

Sulfhydryl groups are one of the most functional groups in proteins and can not only affect the enzymatic activity within the protein but are also closely linked to protein denaturation (23). When the total sulfhydryl content decreases, the disulfide bonds increase (24), which can cause cross-linking and the aggregation of myogenic fibrils, disrupting the structure of the protein (25). Therefore, in this paper, we determined the changes in the total sulfhydryl content of steamed mussels with storage times under different preservation conditions. The results are shown in Figure 1. The total sulfhydryl content of each group decreased with storage time, and the difference between the groups was significant (*p* < 0.05). The fastest decline was seen in the blank group, followed by the preservation agent group, while the sulfhydryl content of the two groups of mussels treated with the electric field decreased more slowly. The sulfhydryl content of the electric field + preservation agent group of mussels decreased at the slowest rate and the group at the end of the storage period was 5.79 × 10^−3^ ± 0.0008 mmol/g. The entire storage period decreased by only 19 mmol/g. The rate of decrease was only 19.46% during the whole storage period, a similar decreasing trend was also reported by Nopianti et al. (26). Compared with other groups, the highest sulfhydryl content of mussels was found in the combined preservation group during the same period of storage. The decrease in total sulfhydryl content of steamed mussels in this study indicates that the preservation agent and LVVFEF reduce the enzymatic coupling reaction of sulfhydryl groups, slow down the rate of oxidative decomposition of sulfhydryl groups and increase the oxidation of sulfhydryl groups into disulfide bonds (27), similar to the results of Wu et al. (28). This indicates that the electric field combined with the preservation agent can inhibit the oxidation of total sulfhydryl groups and protein denaturation in mussels to some extent.

### 3.3. Effects of the LVVFEF combined with compound preservatives on surface hydrophobicity of mussel protein

**Figure 2:**
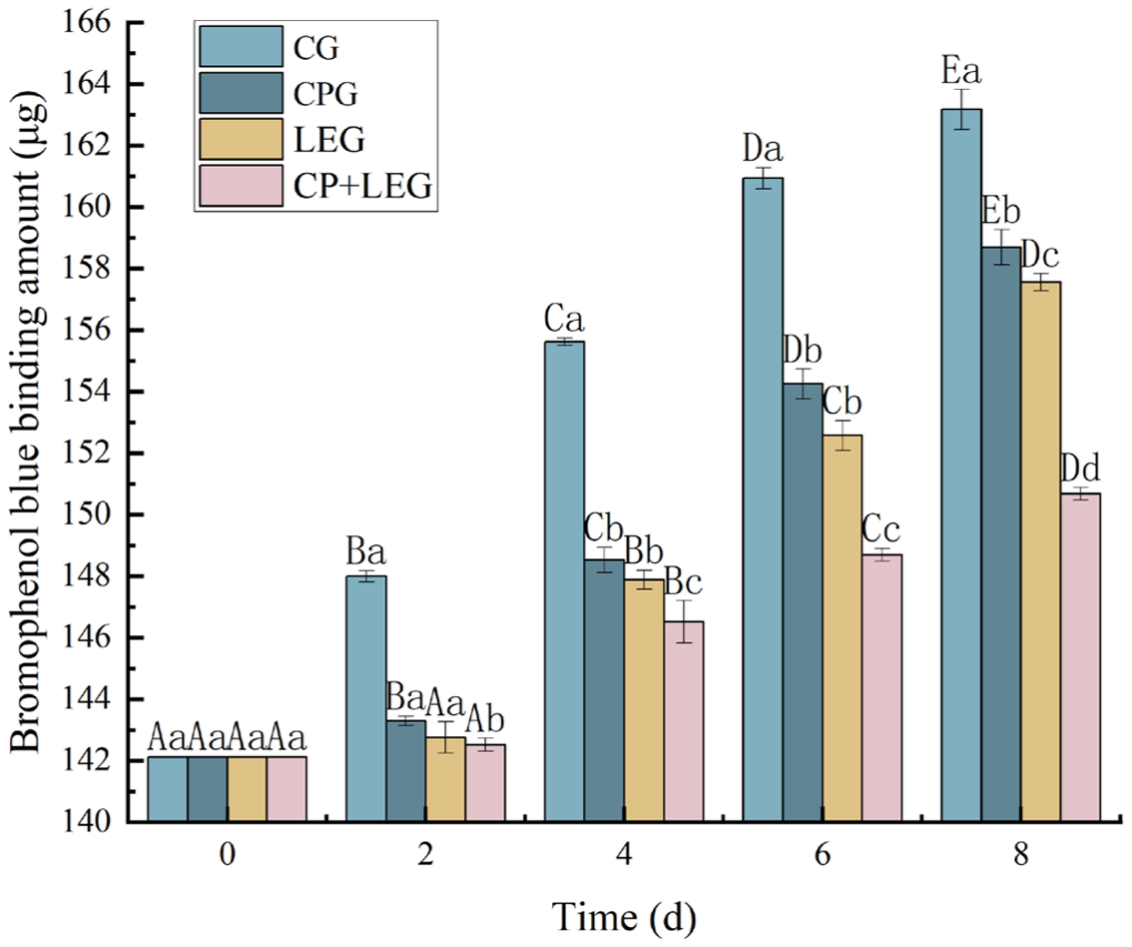
Effect of preservation methods on the hydrophobicity of mussel protein surface. CG, the control group; CPG, the compound preservative group; LEG, the low voltage variable frequency electric field group; CP+LEG, the compound preservative combined with low voltage variable frequency electric field group. At the same storage time, different lowercase letters indicate significant differences between groups; Within the same processing group, different uppercase letters indicate significant differences within the group (*p* < 0.05). Data were obtained as the mean ± standard deviation (*n* = 3).

The surface hydrophobicity of protein molecules is often used to reflect the degree of protein denaturation (29). Therefore, the method of bromophenol blue (BPB) was used to determine the surface hydrophobicity of protein molecules. The principle is to characterize the surface hydrophobicity by the amount of binding between hydrophobic amino acids and bromophenol blue in proteins (30). The variation of protein surface hydrophobicity with storage time for steamed mussels stored at ice temperature and treated by different preservation methods is shown in Figure 2. The hydrophobicity of the mussel protein surface increased with storage time in each group, with significant differences between groups (*p* < 0.05). The CG had the fastest increasing rate, followed by LEG and CPG, and the increasing trend was consistent, and there was no significant difference between groups in the first 6 days (*p* > 0.05). CP+LEG had the lowest rising trend, which was 150.66 μg at the end of storage, which was only 5.67% higher than that at 0 d. The results indicate that the combination of LVVFEF and compound preservatives could prevent the destruction of protein molecules in mussels to a certain extent (31). Based on the determination results of myofibrillar protein content and total sulfhydryl content. In this paper, combined with the results of the determination of total sulfhydryl content, we concluded that the combination of LVVFEF and composite preservation agent can delay the rate of protein oxidation and maintain the spatial structure of the protein, thus achieving the preservation effect, which is consistent with the findings of Harshadrai et al. (32).

### 3.4. Bacterial growth curve

**Figure 3:**
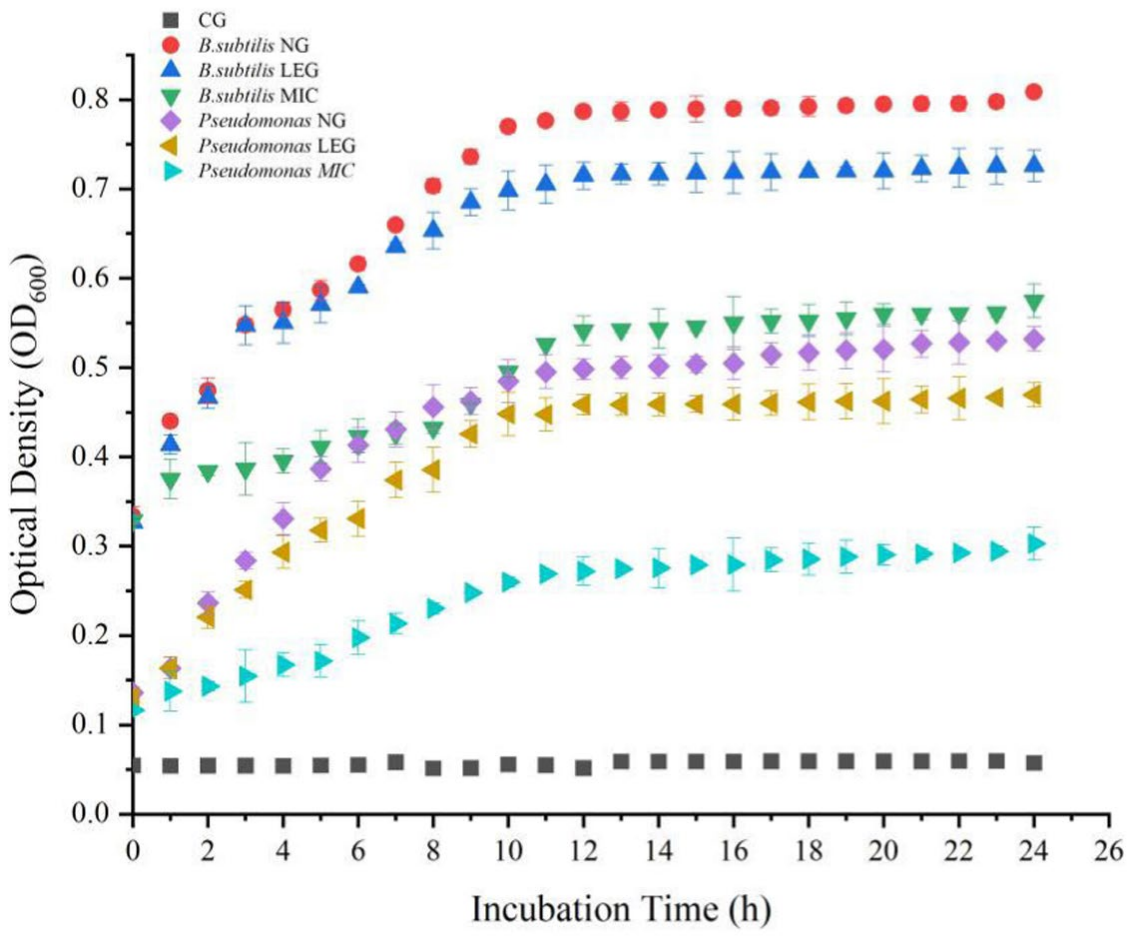
The absorbance value of thallus in OD600 under different conditions. CG, the control group; CPG, the compound preservative group; LEG, the low voltage variable frequency electric field group; CP+LEG, the compound preservative combined with low voltage variable frequency electric field group. Data were obtained as the mean ± standard deviation (*n* = 3).

To understand the effect of different preservation methods on the proliferation of bacteria, this paper measured the growth curves of *Bacillus subtilis* and *Pseudomonas* under the action of LVVFEF and compound preservative. In Figure 3, when the concentration of the compound preservative was MIC, the absorbance values of *Bacillus subtillis* and *Pseudomonas* decreased by 30.16 and 44.58%, respectively, compared with the negative control group. This indicates that the compound preservative had inhibitory effects on the growth of two bacteria and was more effective against *Pseudomonas*. This is because the cell wall of Gram-negative bacteria being thinner. Composite preservative can more easily penetrate the peptidoglycan layer of the cell wall, adsorb the cytoplasm with anions inside the cell, flocculation occurs, the normal physiological activities are disturbed, and causing bacterial death (33). The study of Deng et al. (34) showed that when the preservative was combined with Tremella polysaccharide, lactostreptococcin, and lysozyme it could inhibit the growth of Bacillus, *Pseudomonas,* and Listeriaceae, thus improving the preservation effect of *Penaeus vannamei* in cold storage, which was similar to the results in this study.

In addition, under the environment of a LVVFEF, the growth of the two strains was significantly inhibited. The logarithmic growth period of *Bacillus subtilis* and *Pseudomonas* was 4–8 h, while the logarithmic growth period of the two strains in the negative control group was 4–12 h, indicating that the LVVFEF shortened the growth time of the strain in the logarithmic phase. Compared with the negative control group, the absorbance values of *Bacillus subtilis* and *Pseudomonas* decreased by 9.18 and 10.25%, respectively. This indicates that the inhibitory effect of LVVFEF on *Pseudomonas* was also stronger than that of *Bacillus subtilis.*

### 3.5. Effects of the LVVFEF combined with compound preservatives on cell membrane structures of bacteria

**Figure 4:**
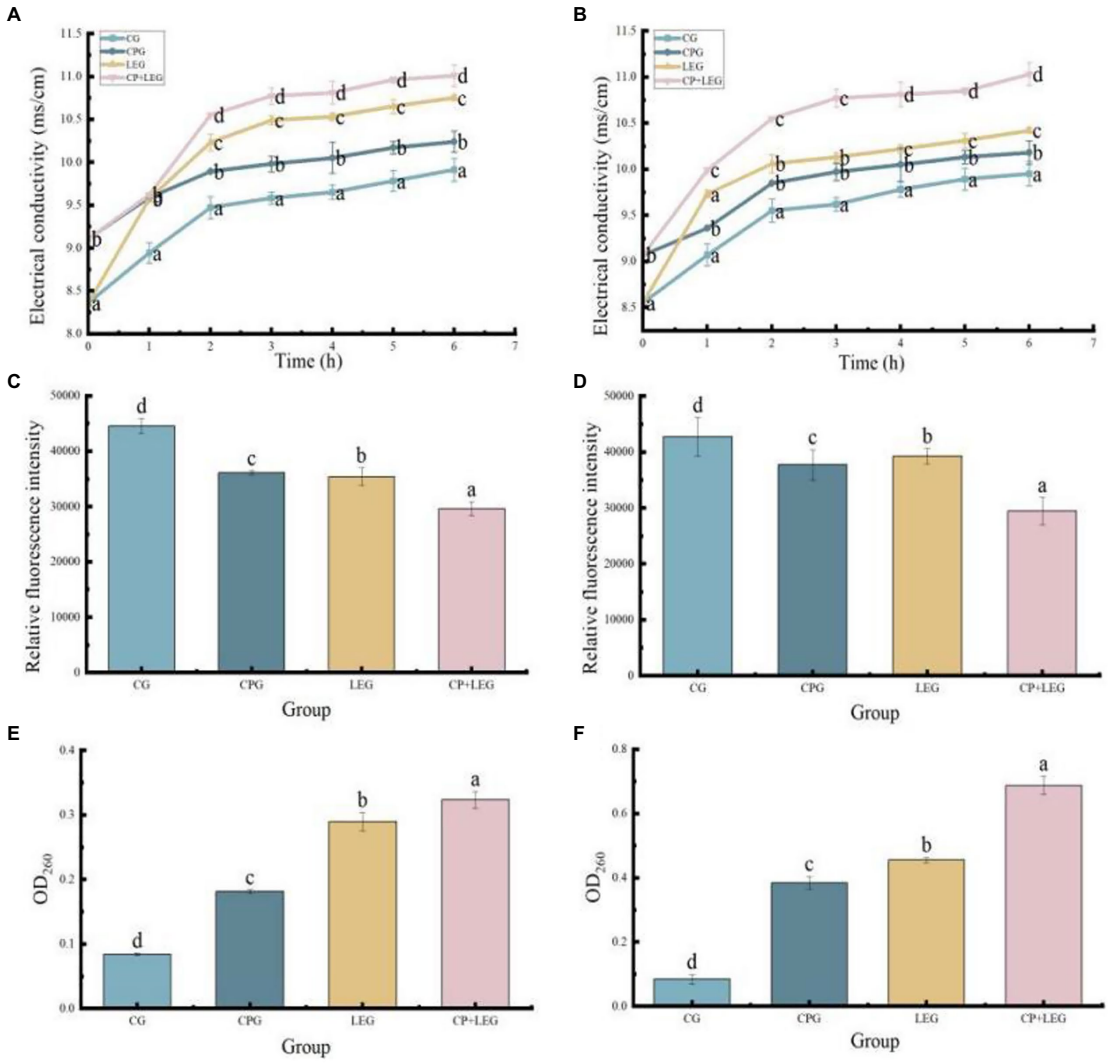
Effects of different preservation methods on the cell membrane of bacteria. CG, the control group; CPG, the compound preservative group; LEG, the low voltage variable frequency electric field group; CP+LEG, the compound preservative combined with low voltage variable frequency electric field group. **(A,C,E)** are *Bacillus subtilis*; **(B,D,F)** are *Pseudomonas*. Different lowercase letters indicate significant differences between groups (*p* < 0.05). Data were obtained as the mean ± standard deviation (*n* = 3).

The cell membrane is the barrier of bacteria. When the cell membrane is destroyed, small molecular ions such as K^+^, Ca^2+^, and Na^+^ in the cell will penetrate the outer membrane, which leads to the enhancement of the electrical conductivity of the bacterial suspension (35) reflecting the change in the permeability of the bacterial cell membrane (36) At the same time, intracellular nucleic acid leaks, and the absorbance value of the conjugated double bond on the nucleic acid structure (-C-C=C-C=C-) at 260 nm can reflect the leakage of nucleic acid (37), which is related to the integrity of the cell membrane. DiBAC4 (3) is an effective sensitive probe, which can produce weak fluorescence. When the cell membrane is depolarized, the dye can enter the cell and bind to cytoplasmic proteins, leading to a change in fluorescence intensity, thus reflecting the type and degree of membrane damage (38). In conclusion, the electrical conductivity, nucleic acid leakage, and membrane potential of bacterial suspension reflect the integrity of cell membrane structure from different perspectives. Therefore, this paper explored the antibacterial mechanisms of a LVVFEF and compound preservatives against *Bacillus subtilis* and *Pseudomonas* by investigating the integrity of the cell membrane structure of bacteria. The results are shown in Figure 4.

**Figure 5:**
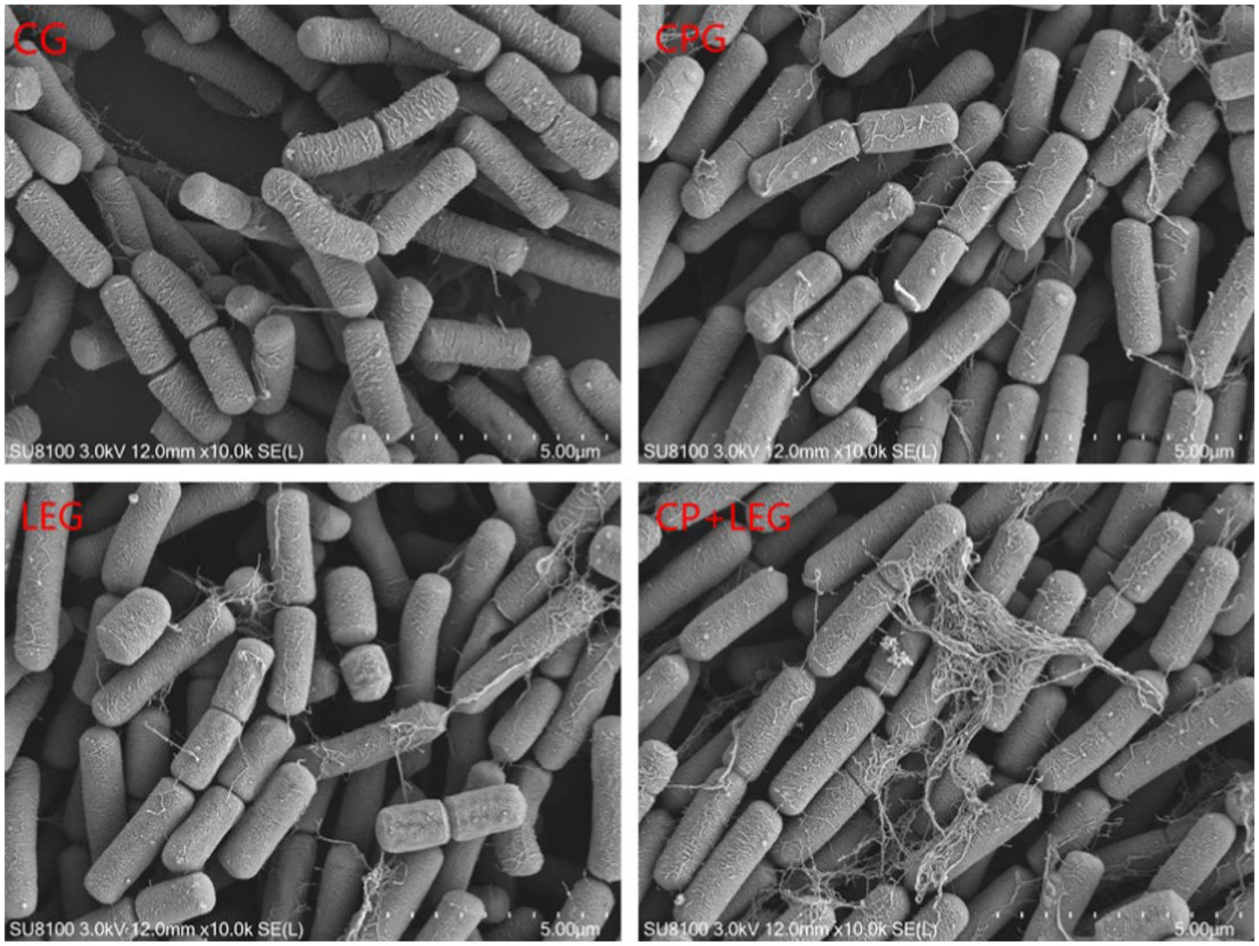
Effects of preservation methods on the morphology of *Bacillus subtilis.* CG, the control group; CPG, the compound preservative group; LEG, the low voltage variable frequency electric field group; CP+LEG, the compound preservative combined with low voltage variable frequency electric field group.

It can be seen in Figures 5A,B, the electrical conductivity of each group increased continuously with the extension of time, and the electrical conductivity of the LEG was always higher than that of the PG and the CG. It has been reported that hyperpolarization is an important type of membrane damage (39). When the cell membrane is hyperpolarized or depolarized, the fluorescent dye will be redistributed inside and outside the cell, and the membrane potential will change. When hyperpolarized, the relative fluorescence intensity decreases, while depolarization increases. As can be seen in Figures 5C,D, the fluorescence values of all treated groups decreased. Analysis of the reason is that after treatment cell membrane permeability increases, membrane potential decreases, ion channels on the bacterial cell membrane are opened or damaged by lysis, the cell ruptures and the contents of the bacterium leak (40). Explained by the outflow of K^+^ in the cell membrane through protein channels on the membrane, increasing the negative charge in the cell and the potential difference (41), and then increasing the transmembrane voltage of the cell membrane, leading to the formation of micropores, beyond the insulation strength of the membrane itself. When the transmembrane voltage exceeds a threshold, the micropores become larger and the permeability of the membrane increases, leading to electrolyte leakage in the cell and death (38). This is also the reason for the increase in electrical conductivity in each group. As can be seen in Figures 5E,F, the OD 260 value of the LEG is higher than that of the CG and the fresh-keeping group, indicating that the damage degree of the bacterial cell membrane is large and the nucleic acid leakage of the bacterial cell is large, which verifies the perforation effect of the LVVFEF on the cell membrane. The results were consistent with the experimental results of membrane permeability and membrane potential.

It also can be seen in Figure 4 that the CPG had a significant effect on the structure of the cell membrane. The electrical conductivity of bacterial suspension treated with compound preservatives was higher than that of the CG, similar to the results of Yang et al. (42). The conductivity of the bacterial suspension was obtained from the interaction of the components of the composite preservative with the cell structure components of the bacteria. Studies have shown that eugenol can destroy the cell wall of the bacterial lipopolysaccharide layer, expose peptidoglycan, and open ion channels. *Lysozyme* can hydrolyze β-1, 4-glycosidic bonds of peptidoglycan in bacterial cell walls (35). *Nisin* through free diffusion from the extracellular membrane into the bacteria body releases protons, interferes with the normal metabolism of bacteria, and accelerates the loss of electrolytes in the cell rate (43), the three have a synergistic effect. Therefore, the loss of charged ions in the cell through the channel led to the charge imbalance inside and outside the cell membrane, the change of membrane potential, and the leakage of intracellular nucleic acid, which explained the why the OD 260 value of the CPG was higher than that of the CG.

In conclusion, CP+LEG had the most significant effect on the cell membrane of bacteria, indicating that LVVFEF and compound preservative had a synergistic effect, which was more likely to cause hyperpolarization of the cell membrane of bacteria, inhibit the metabolic pathway of bacteria, damage the cell membrane structure, resulting in electrolyte and nucleic acid leakage, and eventually lead to death (44).

### 3.6. Effects of the LVVFEF with integrated compound preservatives on bacterial morphology

**Figure 6:**
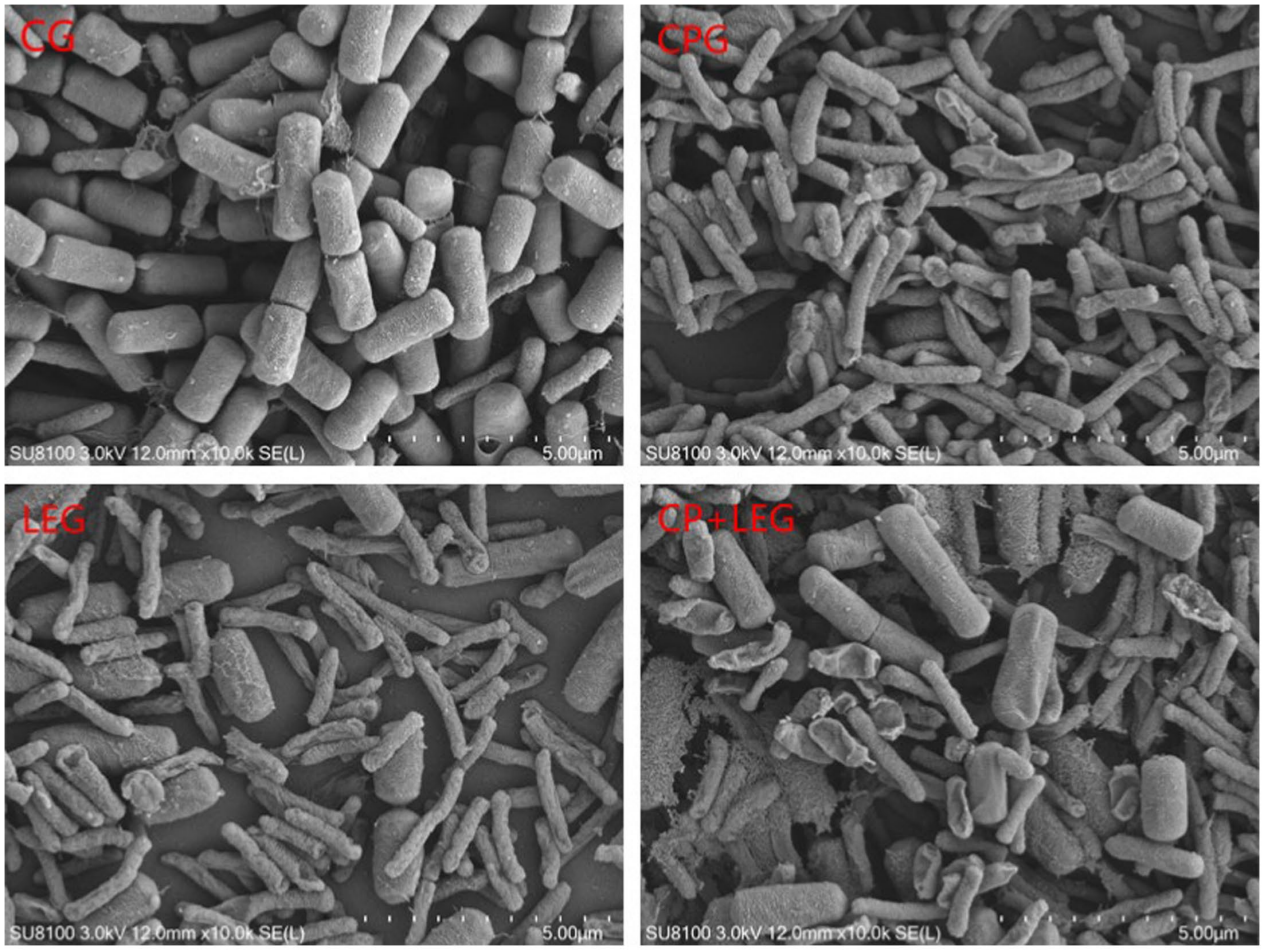
Effects of preservation methods on the morphology of *Pseudomonas.* CG, the control group; CPG, the compound preservative group; LEG, the low voltage variable frequency electric field group; CP+LEG, the compound preservative combined with low voltage variable frequency electric field group.

The effects of the LVVFEF and compound preservatives on the morphology of *Bacillus subtilis* and *Pseudomonas* were observed by scanning with an electron microscope. The results are shown in Figures 5, 6. The CG without any treatment maintained normal thallus morphology. The test bacteria in the blank group without any treatment maintained normal bacterial morphology, that is, the bacteria were rod-shaped, the surface was smooth and flat, the bacterial growth state was good, the bacterial body was full, and there was no shrinkage. The surface of the two bacteria treated by different preservation methods becomes rough, and there are phenomena such as shrinkage, breakage, and leakage of flocs. Compared with the preservative group, the electric field group had a stronger degree of damage to the cell structure of the two bacteria. CP+LEG has the strongest degree of damage to the cell membranes of the two bacteria, especially *Pseudomonas.* There was also a phenomenon of swelling and rupture of some cells. It was once again revealed that the treatment of LVVFEF combined with preservatives had a stronger inhibitory effect on *Pseudomonas* than *Bacillus subtilis.*

## 4. Conclusion

The present study showed that the combination of the preservation agent and the LVVFEF treatment was the best for maintaining the quality of steamed mussels during ice temperature storage and slowing down the rate of protein deterioration during storage compared to the preservation alone or the LVVFEF treatment. The growth curves, cell membrane structure, and electron microscopy results of the strains suggest that the growth rate of *Bacillus subtilis* and *Pseudomonas* was inhibited and the cell membrane structure of the strains was disrupted. The results of the previous experiments showed that the TVB-N value of the mussels in the blank group exceeded the inedible state by the fifth day, while the samples in the combined group did not exceed the standard by the seventh day, indicating that the combination of the freshness preservative and the LVVFEF could prolong the ice temperature storage period of the steamed mussels. This study examined the effects of compound preservative agents combined with low voltage variable frequencies on the preservation mechanisms of steamed mussels being held in ice-temperature storage. By observing the bacterial growth and the structural changes of the cell membrane and comparing the total sulfhydryl content of mussel samples in different groups, we inferred that the combined group could maximize the shelf life of mussels and maintain the quality of steamed mussels. Therefore, this paper lays a theoretical foundation for the application of composite preservatives combined with LVVFEF in food preservation, but also shows wide range of application prospects and value.

## Data availability statement

The original contributions presented in the study are included in the article/Supplementary material, further inquiries can be directed to the corresponding authors.

## Author contributions

KW and HW: methodology, validation, investigation, data curation, formal analysis, visualization, writing—original draft, and review and editing. YW: supervision, resources, and writing—review. CY: investigation and writing—review and editing. YL: data curation. HL and TY: conceptualization, supervision, project administration, resources, funding acquisition, and writing—review and editing. All authors contributed to the article and approved the submitted version.

## Funding

This study was supported by the Zhejiang Zhoushan Archipelago New Area “5313” Action Plan Science and Technology Entrepreneurship Leading Talent Project; National Marine Public Welfare Industry Scientific Research Project (201305016).

## Conflict of interest

The authors declare that the research was conducted in the absence of any commercial or financial relationships that could be construed as a potential conflict of interest.

## Publisher’s note

All claims expressed in this article are solely those of the authors and do not necessarily represent those of their affiliated organizations, or those of the publisher, the editors and the reviewers. Any product that may be evaluated in this article, or claim that may be made by its manufacturer, is not guaranteed or endorsed by the publisher.
